# Using the Animal Model to Accelerate Response to Selection in a Self-Pollinating Crop

**DOI:** 10.1534/g3.115.018838

**Published:** 2015-05-05

**Authors:** Wallace A. Cowling, Katia T. Stefanova, Cameron P. Beeck, Matthew N. Nelson, Bonnie L. W. Hargreaves, Olaf Sass, Arthur R. Gilmour, Kadambot H. M. Siddique

**Affiliations:** * The UWA Institute of Agriculture, The University of Western Australia, Crawley, Western Australia 6009, Australia; † School of Plant Biology, The University of Western Australia, Crawley, Western Australia 6009, Australia; ‡ Norddeutsche Pflanzenzucht Hans-Georg Lembke KG, Hohenlieth, 24363 Holtsee, Germany; § Cargo Vale, Cargo, New South Wales 2800, Australia

**Keywords:** animal model, autogamous plants, pedigree selection, BLUP selection, genomic selection, black spot disease, field peas, GenPred, shared data resource

## Abstract

We used the animal model in S_0_ (F_1_) recurrent selection in a self-pollinating crop including, for the first time, phenotypic and relationship records from self progeny, in addition to cross progeny, in the pedigree. We tested the model in *Pisum sativum*, the autogamous annual species used by Mendel to demonstrate the particulate nature of inheritance. Resistance to ascochyta blight (*Didymella pinodes* complex) in segregating S_0_ cross progeny was assessed by best linear unbiased prediction over two cycles of selection. Genotypic concurrence across cycles was provided by pure-line ancestors. From cycle 1, 102/959 S_0_ plants were selected, and their S_1_ self progeny were intercrossed and selfed to produce 430 S_0_ and 575 S_2_ individuals that were evaluated in cycle 2. The analysis was improved by including all genetic relationships (with crossing and selfing in the pedigree), additive and nonadditive genetic covariances between cycles, fixed effects (cycles and spatial linear trends), and other random effects. Narrow-sense heritability for ascochyta blight resistance was 0.305 and 0.352 in cycles 1 and 2, respectively, calculated from variance components in the full model. The fitted correlation of predicted breeding values across cycles was 0.82. Average accuracy of predicted breeding values was 0.851 for S_2_ progeny of S_1_ parent plants and 0.805 for S_0_ progeny tested in cycle 2, and 0.878 for S_1_ parent plants for which no records were available. The forecasted response to selection was 11.2% in the next cycle with 20% S_0_ selection proportion. This is the first application of the animal model to cyclic selection in heterozygous populations of selfing plants. The method can be used in genomic selection, and for traits measured on S_0_-derived bulks such as grain yield.

Rice, wheat, and other cereals, soybeans, and vegetable-derived oils based on selfing crops account for more than 60% of the world’s food calories for human consumption (Nelson *et al.* 2010). The traditional method for breeding selfing crops may be described as “selfing before crossing,” which delays crossing until after selection of pure lines. This article explores a new method of breeding self-pollinating crops based on application of the animal model to cyclic selection in heterozygous populations, or “crossing before selfing.”

Selection for complex traits in annual autogamous plants normally occurs during or after several generations of selfing, which is followed by crossing among selected near-homozygous parents. Selfing improves the effectiveness of selection by increasing the additive (heritable) variance and decreasing the dominance variance (Wricke and Weber 1986), and selection schemes have been developed to achieve maximum genetic benefit during the selfing phase in autogamous crops (Cornish 1990a,b). Selfing and selection of superior homozygous pure lines is the ultimate goal of breeding in self-pollinating crops (Allard 1999; Wricke and Weber 1986).

However, the delay in crossing until after selfing and selection of superior pure lines imposes limits on breeding of self-pollinating crops. Selection during the selfing process decreases additive genetic variance in the breeding population that cannot be restored when selection is removed, unlike in outcrossing species, and also reduces effective recombination (Cornish 1990a,b). Self-pollinating crop breeding programs tend to have fewer parents in crossing, lower effective population size, and longer generation intervals than animal breeding programs (Cowling 2013). Inbreeding increases the probability that the parents share alleles at pairs of linked loci because of their relatedness and the inbreeding of common ancestors (Hill and Weir 2012). Actual or realized relationship is used in breeding value prediction and is reduced by inbreeding of a parent (Hill 2012; Hill and Weir 2012). Therefore, care must be taken when applying quantitative genetics models, including the animal and genomic selection models, to traditional self-pollinating crop breeding programs.

The animal model exploits information from relatives to estimate breeding values of each related individual in the pedigree (Lynch and Walsh 1998). The pedigree includes individuals with records and their ancestors back to the base population. The data should include records for the selection cohort to avoid selection bias in the current cohort. The pedigree may also include individuals without direct phenotype records (Hill 2012). Breeding values can be estimated for these individuals provided they have measured relatives in the analysis (Walsh and Lynch 2014). In the animal model, selection is based on a selection index known as the predicted breeding value, which combines all information available on the individual and its relatives through a methodology known as best linear unbiased prediction (BLUP) (Henderson 1973).

The annual rate of response to selection (*R*) is related to generation interval (*L*), the accuracy of the selection index (*r*), and the additive genetic standard deviation or standard deviation of breeding values (*σ_A_*) for a given selection intensity (*i*) by the breeders’ equation:

$$R=\frac{i\;\times \;r\;\times \;\sigma_{A}}{L}$$ [1]

The theory behind the breeders’ equation was developed by Lynch and Walsh (1998). Accuracy of the selection index (that is, accuracy of predicted breeding values in the animal model) can be increased by adding more records from relatives in the analysis. Parents, progeny, and full sibs add higher accuracy than collateral relatives (Simm 1998). However, waiting for progeny records increases *L*, whereas including collateral relatives—those from the same generation as the individual—can increase *r* without increasing *L*. Repeated measures of an individual and its relatives, which increase accuracy in the animal model (Simm 1998), are not normally available in segregating S_0_ progeny of annual self-pollinating plants. In theory, self progeny can contribute to the accuracy of the selection index (*r*), but obviously this is not feasible in animal breeding.

For the first time in the animal model, we include records from self as well as cross progeny to improve accuracy of S_0_ predicted breeding values. We combine moderate selection pressure and short generation intervals to accelerate genetic progress, increase effective recombination rates, and minimize genetic drift through large effective population size. Recombination among selected S_0_ plants retains more additive genetic variance in the population, compared with crossing among selected pure lines (Cornish 1990a,b).

Traditional self-pollinating crop breeding programs have long cycle times, historically 6 to 10 yr (Kannenberg and Falk 1995), and this limits the rate of genetic progress for grain yield and other complex traits. Soybean yields of US cultivars released from 1920 to 2000 in maturity groups 4–6 improved by 12.4 kg/ha/yr over 80 yr (approximately 0.5% per yr) (Rogers *et al.* 2015), with generation intervals averaging 10–15 years from historical records (Bernard *et al.* 1988). Yield of US hybrid corn cultivars released from 1920 to 2000 increased by 77 kg/ha/yr from 1930 to 2000 (approximately 1.5% per year), which was mostly based on genetic improvement in inbred parent lines (Duvick *et al.* 2004).

In contrast, some animal breeding programs equal or exceed this rate of annual genetic improvement for economic traits based on best linear unbiased prediction (BLUP) selection (Avendaño *et al.* 2003), underpinned by the animal model. The Meatlinc sheep meat breeding program in the UK achieved genetic progress of 16.5 index units per year (∼15% per year) during the first decade after the introduction of BLUP selection (Avendaño *et al.* 2003). This was achieved with higher effective population size, lower rates of inbreeding, and shorter generation interval than canola (an annual autogamous crop species) in Australia over a similar period (Cowling 2013). Other examples of acceleration in genetic improvement following the introduction of BLUP selection include dairy milk production (Van Doormaal and Kistemaker 2003) and beef carcass value (Bouquet *et al.* 2010).

BLUP methods in autogamous crops are normally based on fixed lines with phenotypic records restricted to the current generation (Beeck *et al.* 2010; Cullis *et al.* 2010). Selection in the animal model introduces bias, but BLUP analyses accommodate this if all data used in previous selection decisions are included in the analysis (Piepho and Möhring 2006), and the relationship matrix includes all ancestors back to base individuals in the pedigree (Piepho *et al.* 2008). This is difficult to achieve in self-pollinating crops based on traditional approaches to breeding. Typically, generation intervals are 6 yr or longer (Cowling 2013), and records from previous generations are not available.

We propose a combined analysis across cycles for self-pollinating crop plants, based on the animal model. For the first time, the combined analysis includes relationship information from self progeny in the analysis. Selfing of each S_1_ or S_2_ individual in the relationship matrix is explicitly declared through identical male and female parents (Figure 1, Supporting Information, Table S3). The combined analysis is based on two cycles of S_0_ (F_1_) recurrent selection in field pea (*Pisum sativum* L.) for resistance to ascochyta blight [major pathogen *Didymella pinodes* (Berk. & Blox.) Petrak] (Khan *et al.* 2013). *P. sativum* is the highly self-pollinating annual plant species used by Mendel to demonstrate the particulate nature of inheritance.

**Figure 1:**
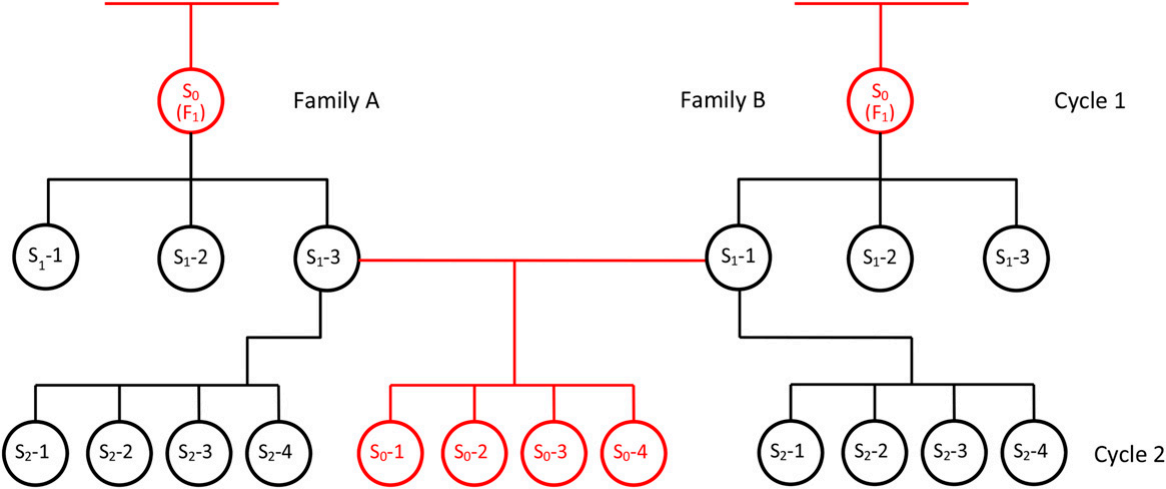
A portion of a pedigree over two cycles of selection in the self-pollinating plant version of the animal model. Circles represent hermaphroditic plants in an annual autogamous crop. Pedigree relationships typically found in the animal model are represented by red relationship lines and circles. The plant model includes selfing between cycles (black relationship lines and circles). S_0_ plants are phenotyped in each cycle and selected on the basis of predicted breeding value from a combined analysis across cycles based on a linear mixed model. Several S_1_ parent plants are grown for crossing between family A and family B (only one cross shown here). S_0_ phenotypes may be measured directly on the individual, such as ascochyta blight score in pea (this article), or indirectly on S_0_-derived bulks, such as seed oil content or grain yield. S_2_ plants are available for further selfing and selection of pure lines, following traditional methods.

The statistical model for the combined analysis across cycles is based on the linear mixed model equation [2] of Beeck *et al.* (2010), who analyzed yield and seed oil content in 19 canola (*Brassica napus*) trials over 2 yr, incorporating pedigree information. Correlation of additive, nonadditive, and total genetic affects across trials, as presented by Beeck *et al.* (2010) and Cullis *et al.* (2010), is equivalent to correlation across selection cycles in our model.

In our experiments, S_0_ plants are selected for resistance to ascochyta blight in the field and the next cycle begins by crossing S_1_ plants harvested from selected S_0_ individuals. Cross progeny (S_0_) and self progeny (S_2_) are saved from each parent S_1_ plant. These S_2_ and S_0_ progeny are augmented half-sibs related by a common S_1_ parent plant, and both provide records to improve accuracy of S_0_ prediction (Figure 1). Pure-breeding ancestors are used for replication within experiments (cycles) and genotypic concurrency across experiments (cycles).

Resistance to ascochyta blight is a complex trait with low heritability (Khan *et al.* 2013), but sources of partial resistance have been identified (Wroth 1999; Beeck *et al.* 2006; Adhikari *et al.* 2014), and moderate broad-sense heritability of 0.63 was reported on a single plant basis (Beeck *et al.* 2008). Annual selection cycles can be achieved in *P. sativum* if crossing among S_1_ parent plants occurs in the contra season. A combined analysis across cycles should increase accuracy of selection and accelerate response to selection through a major reduction in generation interval compared with traditional methods of breeding complex traits in self-pollinating crops.

If successful, our model could be extended to traits measured on S_0_-derived bulks, such as grain quality or yield, and genomic relationship data may complement or replace pedigree relationship data to improve the accuracy of the selection index, as reported in the animal model (Hayes *et al.* 2009). The combined analysis of data across generations, as proposed in our model, will increase the validity of genomic selection models for self-pollinating crops, which have not yet included training and selection candidates across generations (Jonas and De Koning 2013). The model is readily incorporated into traditional breeding programs of self-pollinating crops, where it will supply preselected S_2_-derived lines for further inbreeding and development of pure lines.

## Materials and Methods

### Plant material and crossing

The field pea trial grown in 2010, cycle 1, included 959 segregating S_0_ individuals representing 115 crosses among 76 diverse parent genotypes. The parent genotypes of *P. sativum* were derived from a recurrent selection program (Beeck *et al.* 2008) with founder lines including progeny of wild × domestic crosses, landraces from Greece, and cultivars that varied significantly in resistance to ascochyta blight (Beeck *et al.* 2006). Other plants grown in 2010 were F_2_ progeny of crosses between pure-line Australian and European (labeled “NPZ-” and “G-”) cultivars and Australian and Chinese (labeled “CCC-”) landraces. Founder genotypes in the base population, Australian cultivars and Chinese landraces were used as replicated controls. Parent plants used in crossing to generate cycle 1 and cycle 2 were not pure lines but segregating S_0_-derived S_1_ selections, which generated segregating S_0_ progeny. Prior to cycle 1, heterozygous parents (F_2_ genotypes) from crosses of pure line parents were selected for crossing. Genotype names and data for cycle 1 are shown in Table S1.

The plants grown in 2010 were assessed for ascochyta blight resistance and 102 of the 959 cycle 1 S_0_ individuals were selected for harvest of S_1_ seed in the field. The pedigree of the selected S_0_ individuals from cycle 1 included 26 of the 115 cycle 1 crosses and 33 of the 76 cycle 1 parents. The cycle 2 crossing design was based on 53 pair-wise combinations among the cycle 1 S_0_ selections, avoiding, when possible, common grandparents. Up to three S_1_ plants were grown per S_0_ selection, and up to five crosses were made among the S_1_ plants within each pair-wise combination. A total of 430 viable S_0_ seeds were harvested from the 53 pair-wise combinations for sowing in cycle 2. Crossing occurred in either direction according to bud maturity, because *P. sativum* is hermaphroditic and maternal effects were not apparent for ascochyta blight resistance (Wroth 1999), and S_1_ plants were occasionally used more than once. Genotype names and data for cycle 2 are shown in Table S2, and the pedigree relationships of a cycle 2 S_0_ progeny (genotype 15 in Table S6) are fully described in Table S3 and Table S6. The pedigree and phenotypic records for genotype 15 from Table S6, showing crossing and selfing in the ancestry, and records from a sample of relatives including full-sib, half-sib, augmented half-sib, and S_2_ self progeny of S_1_ parent plants, are summarized below following the style of Henderson (1973) in Table 1.

**Table 1:** Pedigree and phenotypic records for genotype 15

Genotype	Dam	Sire	Disease Score Cycle 1	Disease Score Cycle 2
1	0	0		9.0
2	0	0	9.0	9.8
3	0	0	10.0	8.5
4	0	0	10.0	8.0
5	0	0	10.5	9.0
6	0	0	14.0	
7	0	0	12.0	11.1
8	2	1		
9	4	3		
10	9	8		
11	6	5	6	
12	10	7	11	
13	11	11		
14	12	12		
**15**	**14**	**13**		**8**
16	12	12		
17	14	13		11
24	16	13		14
31	14	14		12
35	13	13		9

**Figure 2:**
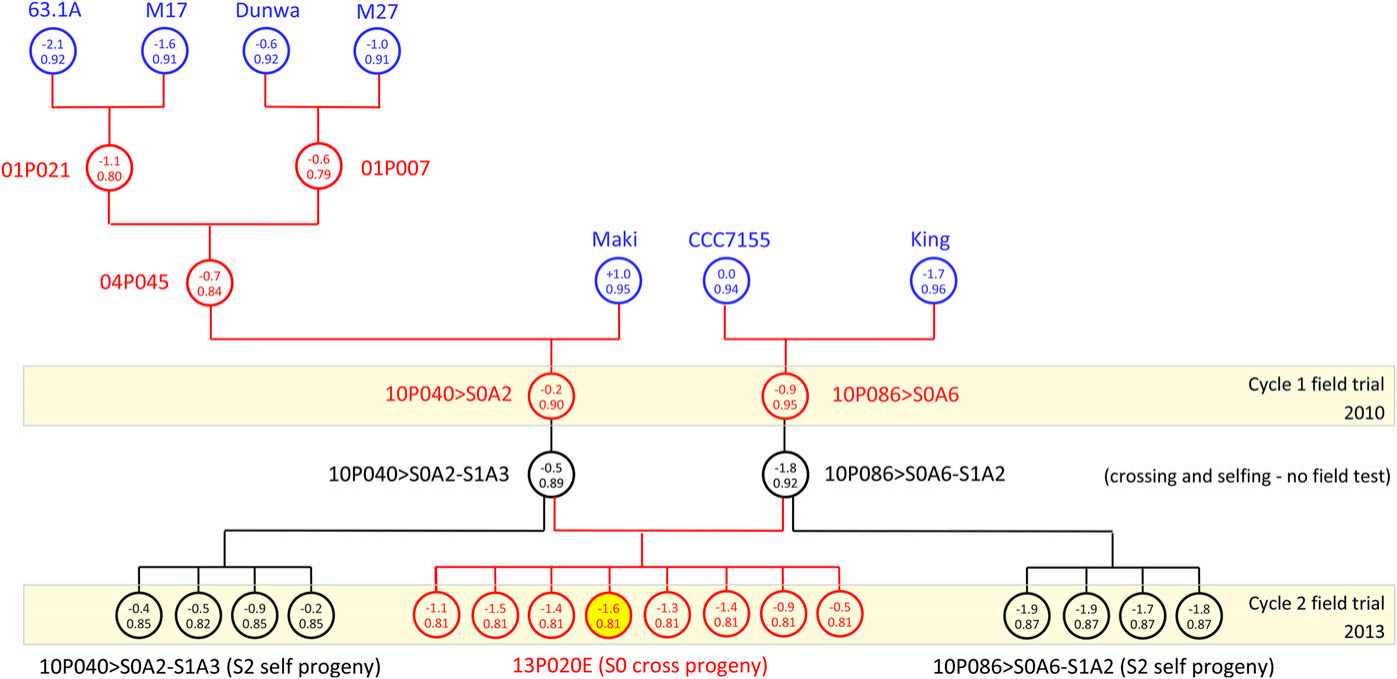
Predicted breeding values (upper half of circles) and accuracy (lower half of circles) for ascochyta blight score for one family of S_0_ and S_2_ sibs based on cycle 2 predictions. Relatives in the pedigree include S_1_ parent plants and S_2_ selfs of parent plants (black circles), replicated pure lines, and founder varieties in the base population (blue circles). The fourth S_0_ progeny highlighted in yellow (13P020E>S0A4) is chosen for crossing to start the next cycle when the selection proportion is 25%, and several resistant S_2_ progeny are available for further selection and production of pure lines. The relationships of this genotype with its ancestors and some of its self and cross progeny are shown in Table S6 (genotype 15).

Pedigree and phenotypic records for genotype 15 from Table S6, defined as progeny 13P020E>S0A4 in Figure 2, showing crossing and selfing in the ancestry, and records from a sample of relatives including full-sib, half-sib, augmented half-sib, and S_2_ self progeny of S_1_ parent plants, are summarized following the style of Henderson (1973).

### Design and management of field trials

The field trials for cycle 1 and cycle 2 were designed as partially replicated (p-rep) trials (Cullis *et al.* 2006) using DiGGeR (Coombes 2009) with the default spatial model. Plots were single plants spaced 1 m apart in rows and columns, with 40 columns by 30 rows in cycle 1 grown in 2010, and 20 columns by 70 rows in cycle 2 grown in 2013. A total of 1139 and 1077 early generation progeny plants were tested in cycle 1 and cycle 2, respectively (Table 2). Replication and concurrency of genotypes across experiments were provided by pure lines including founder lines, Australian cultivars, and Chinese landraces (Table 2, Table S1, Table S2).

**Table 2:** Number of genotypes, replicates, and plots of early generation progeny and control varieties contributing to cycle 1 (2010) and cycle 2 (2013) field experiments

	Cycle 1			Cycle 2		
	Genotypes	Replicates	Plots	Genotypes	Replicates	Plots
Early generation progeny	1139			1077		
S_0_ progeny	959	1	959	430	1	430
S_2_ selfs of parent plants	133	1	133	609	1	609
F_2_ progeny	47	1	47	38	1	38
Control varieties	30			15		
Control varieties 2 reps	29	2	58			
Control varieties 3 reps	1	3	3			
Control varieties 11 reps				1	11	11
Control varieties 14 reps				1	14	14
Control varieties 22 reps				5	22	110
Control varieties 23 reps				4	23	92
Control varieties 24 reps				4	24	96
Total varieties tested	1169			1092		
Total plots			1200			1400

Fourteen of the control varieties provided genotypic concurrence across cycles. S_0_ progeny have heterozygous parents; F_2_ progeny have fixed line parents.

Pea seeds were germinated in the glasshouse in early May (late autumn) and transplanted into the field in late May 2010 (cycle 1) and 2013 (cycle 2) at The University of Western Australia field station, Shenton Park, Western Australia. Helena, a field pea variety susceptible to ascochyta blight, was sown along the edges of the trial, and ascochyta blight-infected pea straw was spread across the field trials 1 wk after transplanting to encourage an epidemic of ascochyta blight. The dry pea straw was collected at the end of the previous growing season from susceptible varieties grown at Medina, Western Australia. Each plot was labeled with range, row, and genotype name.

Field trials were managed according to standard recommendations for field pea crops in Western Australia (White *et al.* 2005). Disease developed under natural rainfall conditions and no supplemental irrigation was required. Dry seeds were harvested from senesced plants in October or November.

### Assessment of severity of ascochyta blight

Severity of ascochyta blight was assessed on each plant in mid July by counting from the first leaf node upwards the number of nodal leaves with more than 50% leaf infection, based on visual comparison with guides used by James (1971) to assess leaf spot of red clover. This count was defined as the ascochyta blight score (ABS), with low ABS representing greater resistance, and high ABS representing greater susceptibility.

### Best linear unbiased prediction

All statistical models were fitted using statistical software ASReml-R (v. 3.0) (Butler *et al.* 2009), which produces residual maximum likelihood (REML) estimates of the variance parameters and BLUP of the random effects.

Spatial variation in ABS across each trial was assessed following the mixed model approach described by Gilmour *et al.* (1997) and Stefanova *et al.* (2009). The modeling process began by fitting a first-order separable autoregressive residual model (AR1 × AR1) to identify local trends along rows and columns; the model was then revised to include significant linear trend and/or random range/row effects. The effects of local and trend spatial variation were assessed using a plot of residuals, the sample variogram, row and column faces of the empirical variogram, and REML likelihood ratio tests. Possible outliers in ABS were assessed and removed.

A pedigree file was prepared including all founders and ancestors of genotypes tested in cycles 1 and 2, including those without records (Table S3). The inbreeding of replicated ancestor lines or cultivars was indicated as the number of generations of selfing in the “Fgen” field as required by ASReml. Otherwise each selfed generation was explicitly declared with identical female and male parents (Table S3). Both additive (predicted breeding values correlated according to the relationship matrix) and nonadditive (uncorrelated) genetic effects were included in the mixed model for ABS, with cycle-specific variance components.

Three genetic models were fitted. The base model ignored genetic relationships and covariances between cycles. The second model included genetic relationships but ignored covariances between cycles. The full model included the additive and nonadditive genetic covariances between cycles. Variance components were estimated for additive and nonadditive effects in each cycle and for the covariance between cycles, and these were used to calculate the correlation of predicted ABS breeding values across cycles. Narrow-sense heritability for ABS was calculated from the full model as the ratio of the additive variance component divided by the sum of additive, nonadditive, and error variance components in each cycle.

ASReml-R scripts for the three models are provided in Table S4.

### Accuracy of predicted breeding values

The accuracy of predicted breeding values (*r*) is the correlation between the true and predicted breeding values and is sometimes reported as reliability, the squared correlation (*r*^2^) (Mrode 2005). The accuracy *r_i_* of the predicted breeding value for the *i*-th genotype was calculated according to Gilmour *et al.* (2009) for the animal model, namely

$$r_{i}=\sqrt{1-\frac{s_{i}^{2}}{(1+f_{i})\sigma_{A}^{2}}}$$ [2]

where *s_i_*^2^ is the prediction error variance for the *i*-th genotype, *σ_A_*^2^ is the additive genetic variance, *f_i_* is the inbreeding coefficient for the *i*-th genotype, and (1 + *f_i_*) is the diagonal element of the relationship matrix *A*. ASReml-R script for accuracy is provided in Table S4.

## Results

### Analysis of ABS across cycles and models

The three genetic models are compared in Table 3 based on the same spatial model. Partitioning the genetic variance into additive and nonadditive (uncorrelated) components using the pedigree-based relationship matrix significantly increased the log likelihood of the mixed model for ABS from the base model to second model (Table 3). The significant, albeit small, increase in REML likelihood from the second model to the full model (Table 3) is almost all due to the covariance of additive effects and indicates an advantage from analyzing the two cycles together.

**Table 3:** Analysis of two cycles of selection for resistance to ascochyta blight in *Pisum sativum*

	Base Model	Second Model	Full Model
REML LogLikelihood	−3458.5	−3301.0	−3296.6
Significance of change between models		*P* < 0.001	*P* = 0.012
Fixed effects: Incremental Wald F statistic with 1 df			
Site	33.3*^c^*	2.9 ns	0.4 ns
Linear row cycle 1	3.2 ns	3.5 ns	3.9*^a^*
Linear row cycle 2	102.4*^c^*	105.2*^c^*	105.4*^c^*
Linear range cycle 1	126.9*^c^*	41.8*^c^*	44.9*^c^*
Linear range cycle 2	10.9*^c^*	9.6*^b^*	9.4*^b^*
Variance components for random effects:			
Residual components			
Variance cycle 1	4.730 ± 1.367	3.774 ± 1.015	3.802 ± 1.021
Range autocorrelation	−0.053 ± 0.047	−0.063 ± 0.050	−0.062 ± 0.049
Row autocorrelation	0.004 ± 0.046	−0.013 ± 0.049	−0.014 ± 0.048
Variance cycle 2	3.715 ± 0.301	3.603 ± 0.279	3.533 ± 0.267
Range autocorrelation	0.167 ± 0.046	0.167 ± 0.040	0.173 ± 0.040
Row autocorrelation	0.061 ± 0.044	0.062 ± 0.038	0.064 ± 0.039
Other spatial components			
Row effects cycle 1	0.109 ± 0.075	0.114 ± 0.068	0.118 ± 0.069
Range effects cycle 2	0.124 ± 0.076	0.096 ± 0.061	0.096 ± 0.061
Blocks cycle 1	0.000 ± N/A	0.039 ± 0.111	0.033 ± 0.102
Blocks cycle 2	0.013 ± 0.286	0.000 ± N/A	0.000 ± N/A
Total genetic effects			
Variance cycle 1	1.802 ± 1.367		
Variance cycle 2	2.975 ± 0.408		
Additive genetic effects			
Variance cycle 1		1.864 ± 0.425	2.098 ± 0.450
Variance cycle 2		2.336 ± 0.408	2.286 ± 0.392
Correlation			0.823 ± 0.111
Nonadditive genetic effects			
Variance cycle 1		1.046 ± 1.014	0.970 ± 1.019
Variance cycle 2		0.537 ± 0.370	0.673 ± 0.352
Correlation			0.673 ± 0.924
Narrow-sense heritability			
Cycle 1			0.305
Cycle 2			0.352
Average accuracy of predictions			
Cycle 1	0.562	0.894	0.815
Cycle 2	0.696	0.807	0.834

Based on ascochyta blight score (ABS) on single-spaced plants in partially replicated field experiments in cycle 1 (2010) and cycle 2 (2013). Three genetic models were fitted. The base model ignored genetic relationships and covariances between cycles. The second model included genetic relationships but ignored covariances between cycles. The full model included the additive and nonadditive genetic covariances between cycles. The models included fixed effects (experiment, linear row, and linear range effects) and random effects (additive and nonadditive genotype effects, genotype by environment effects, blocking structure, random row effects, and range and row correlations from the AR1:AR1 spatial model). Narrow-sense heritability is estimated in cycles 1 and 2 from the full model, ignoring the fact that the residuals are correlated, and average accuracy of predictions was calculated for genotypes present in each cycle for each model. ns, not significant; N/A, not available.

a 0.01 < *P* < 0.05.

b 0.001 < *P* < 0.01.

c *P* < 0.001.

The overall level of disease was similar in the two experiments, with ABS 11.2 and 10.2 leaves in cycle 1 and cycle 2, respectively. This is reflected in the nonsignificant effect of site in the full model (Table 3). There were significant linear row spatial effects in cycle 2, linear range effects in both cycle 1 and cycle 2 trials, significant random row effects in cycle 1, and random range effects in cycle 2 (Table 3). Hence, spatial analysis and relationship information was very important to reduce unexplained error and improve accuracy of estimation of genetic effects in ABS in these field trials.

The full model showed significant additive components for ABS in cycles 1 and 2, and significant nonadditive components in cycle 2, but additive was always two-fold or three-fold larger than the nonadditive component (Table 3). Narrow-sense heritability for ABS from the full model was 0.305 in cycle 1 and 0.352 in cycle 2. The fitted genetic correlation of breeding values (additive effects) across cycles was 0.82 ± 0.11, and not significant for the nonadditive effects, but there were only 14 genotypes in common plus the links induced by the 102 S_0_-derived genotypes from cycle 1 used as parents for cycle 2.

Partial replication within experiments, provided by pure lines, many of which were ancestors or founders, permitted estimation of additive effects for all genotypes from each experiment (cycle) and nonadditive effects in cycles where they occurred. The improvement from the second model to full model is small because most of the information on the 102 S_0_-derived S_1_ genotypes used as parents in cycle 2 comes from the cycle 2 data.

### Accuracy of predicted breeding values and response to selection

Average accuracy of prediction increased from the base, second to full model (Table 3). In the full model, predicted breeding values for ABS on 430 S_0_ progeny in cycle 2 ranged from −3.5 to +1.7 and the accuracy of predicted breeding values from equation [2] ranged from 0.731 to 0.854 (average 0.805) (Table 4, Table S5). The average accuracy of predicted breeding values of 160 S_1_ parent plants, for which no records were available, was 0.878 (Table 4). High accuracy was also achieved on 575 S_2_ self progeny of parent plants (average 0.851) (Table 4).

**Table 4:** Mean and range of BLUP of predicted breeding values and accuracy of predictions for ascochyta blight score*^a^*

		BLUP			Accuracy		
	No. Individuals	Low	Mean	High	Low	Mean	High
Cycle 2 S_0_ progeny	430	−3.473	−0.831	1.668	0.731	0.805	0.854
Cycle 2 S_1_ parent plants	160	−3.929	−0.838	2.431	0.729	0.878	0.916
Cycle 2 S_2_ selfs of parent plants	575	−3.959	−0.826	3.192	0.733	0.851	0.873

a Data were generated from the full model for cycle 2. Phenotypic records were available for S_0_ progeny and S_2_ selfs of parent plants in cycle 2, but not for S_1_ parent plants.

The SD of additive effects (predicted ABS breeding values) for 430 S_0_ progeny in cycle 2 was 1.01. With a selection proportion of 20% of S_0_ lines (selection intensity of 1.40) and average accuracy of predicted breeding values 0.805, the response to selection based on equation [1] in the next cycle is forecast to be −1.1 leaves or −11.2% of average ABS in cycle 2 (10.2 leaves).

Predicted breeding values and their accuracy varied among S_0_ progeny of one cross, 13P020E, evaluated in cycle 2 (Figure 2). The fourth S_0_ progeny of cross 13P020E with predicted breeding value −1.6 was selected for crossing when the selection proportion was 25%. Further investigation revealed that the S_1_ parent plants of 13P020E had the lowest predicted breeding values of all possible S_1_ parent plants in this pair-wise combination. Parent plant 10P086 > S0A6-S1A2 had a predicted breeding value of −1.8 (Figure 2), but its sister parent plant 10P086 > S0A6-S1A1 was significantly more susceptible, with predicted breeding value of +0.3 (Table S5). As a result, no other S_0_ progeny of crosses 13P020A, 13P020B, 13P020C, or 13P020D were within the 25% selection proportion.

## Discussion

Selection for resistance to ascochyta blight on single plants of *P. sativum* was based on a simple measurement (ABS) that was accurately scored by different assessors in 2010 and 2013. ABS was a quantitative trait with low heritability in cycle 1 (0.305) and cycle 2 (0.352), but predicted breeding value was expressed consistently over the years (correlation of additive genetic effects across cycles 0.82) (Table 3). Following the logic of Falconer and Mackay (1996) with respect to genotype by environment interaction, ABS measured in cycle 1 and cycle 2 is not one character but two, and the power of the combined multivariate analysis benefits is increased by the close correlation between the two.

Selection for ABS in the recombination phase of breeding self-pollinating crops will complement, not replace, selection on pure lines in field plots (Adhikari *et al.* 2014). Low ABS counts are a measure of resistance to this disease, but low counts could also reflect small plants, so a future improvement may include counting the total number of leaf nodes as a potentially useful covariate for ABS. Flowering date may also have been a useful covariate as previous results indicated that ascochyta blight disease severity was lower in later flowering types (Beeck *et al.* 2008). The model can accommodate improvements such as these in future cycles of selection.

A traditional analysis of these data are represented by our base model, where each plant is evaluated solely from its own assessment. Our results show large benefits from adding information from relatives in the second model and further improvements by including additive genetic covariances between cycles in the full model. This demonstrates value in applying the animal model to S_0_ recurrent selection in the self-pollinating annual crop, *P. sativum*, especially when self progeny of S_1_ parent plants are included in the analysis. BLUP analyses accommodate bias introduced by selection if all data used in previous selection decisions are included in the analysis (Piepho and Möhring 2006). Relationship and phenotypic records are available in cycles 1 and 2 for selected and nonselected self and cross progeny (Table S1, Table S2), so this plant model conforms to the requirements of the animal model, and unbiased BLUP selection may continue into the future by combining data with previous cycles of selection. To the best of our knowledge, this is the first application of the animal model to cyclic selection in heterozygous populations of selfing plants.

Frey and Holland (1999) reported significant increases in oil content over several cycles of S_0_ recurrent selection in oats, and Zhang *et al.* (2015) for wider seedling leaves in wheat, but their methods did not incorporate BLUP selection based on pedigree information. Our experiments show that, with a relatively small investment in records management and analysis, it is possible to derive great benefits from BLUP-based S_0_ recurrent selection in self-pollinating crops. Benefits of BLUP selection in animals include increased response to selection as a result of more accurate breeding values and shorter generation intervals (Simm 1998; Avendaño *et al.* 2003; Van Doormaal and Kistemaker 2003). In some cases, annual genetic progress in animals under BLUP selection is faster and inbreeding rates are lower than in typical self-pollinating crop breeding programs (Kannenberg and Falk 1995; Cowling 2013).

Narrow-sense heritability of resistance to ascochyta blight in *P. sativum* reported in this study (0.30–0.35) is consistent with moderate broad-sense heritability (0.63) on a single plant basis reported previously (Beeck *et al.* 2008). With moderate selection pressure (20%), the forecasted response to selection for resistance in the next cycle is 11.2% based on equation [1]. In *P. sativum*, annual cycles of S_0_ recurrent selection are possible by crossing S_1_ parent plants in the contra season, so it is possible to achieve 11.2% improvement in the next 12 months. This predicted response to selection on an annual basis is much higher than achieved previously in breeding for resistance this disease (Khan *et al.* 2013).

It is not possible, in the case of ABS in annual *P. sativum*, to cross with phenotyped S_0_ plants, because they naturally self-pollinate in the field at the time the plants are being rated for ABS. Therefore, crossing was performed in the glasshouse on the S_1_ self progeny of selected S_0_ plants (Figure 1), and no ABS records are available for S_1_ parent plants. The animal model may include individuals in the pedigree without records (Hill 2012), provided such individuals have measured relatives in the analysis (Walsh and Lynch 2014). S_1_ parent plants, which were intercrossed to start cycle 2, had high accuracy of predicted breeding values (average 0.878) as a result of records from self (S_2_) and cross (S_0_) progeny in cycle 2 (Figure 2, Table 4). Records from full-sibs, half-sibs, parents, S_0_ individuals, and collateral relatives including S_2_ progeny of parent plants were valuable to increase accuracy of the predicted breeding values of S_0_ progeny in cycle 2 (average 0.805). This is the first time, to our knowledge, that self progeny have contributed relationship and phenotypic information in the animal model (Figure 1, Figure 2).

An opportunity exists to improve the efficiency of BLUP selection in this model by replacing pedigree relationship information with the realized relationship matrix from genome-wide markers (Hayes *et al.* 2009). Genomic information may be useful to select putative resistant S_1_ plants based on markers trained in previous cycles of selection, before they are used in crossing. Several S_1_ self progeny from selected S_0_ plants were used in crossing, and segregation among these S_1_ genotypes resulted in some poor parents. For example, parent plant 10P086>S0A6-S1A2 had a predicted breeding value of −1.8 ABS (Figure 2), but its sister parent plant 10P086>S0A6-S1A1 was significantly more susceptible (+0.3 ABS) (Table S3), and this was not known until the next cycle of selection based on self and cross progeny records. This is inefficient compared with preselection of S_1_ parent plants for high genomic predicted breeding value, based on markers trained over previous cycles of selection as proposed by Jonas and De Koning (2013).

The plant version of the animal model may be adapted to traits measured on S_0_-derived seed bulks, such as grain quality or yield. At least 1 yr would be added to the selection cycle to obtain yield records, but the generation interval would be much shorter than traditional breeding methods for yield in selfing crops. The plant model is a “low-tech” improvement in traditional plant breeding with potentially large benefits (Snape 2004), but it can readily incorporate new technologies such as genomic selection in self-pollinating crops (Heffner *et al.* 2009, 2011; Nakaya and Isobe 2012; Heslot *et al.* 2015).

The application of genomic selection to inbreeding crops is inhibited by population structure and lack of polymorphisms (Desta and Ortiz 2014). A “select-recombine-self” method was proposed for early generation genomic selection within a biparental population of a self-pollinating crop by Bernado (2010), and this formed the basis of a model for genomic selection for stem rust resistance in wheat (Rutkoski *et al.* 2011). In traditional selfing methods, genomic selection may reduce the number of generations of selfing without sacrificing selection gains (McClosky *et al.* 2013). However, none of these models integrated training and selection candidates across generations as proposed by Jonas and De Koning (2013). The design of the breeding scheme is vital for long-term genetic gain and for exploiting the benefits of genomic information in animal or plant breeding (Henryon *et al.* 2014). We have used the animal model for successful prediction across generations of a self-pollinating crop, and the model will readily accommodate genomic data. This plant model should therefore access many of the theoretical benefits of genomic selection in animals (Goddard and Hayes 2009), cross-pollinating forage crops (Resende *et al.* 2013) and tree crops (Resende *et al.* 2012). Accuracy of selection is expected to increase when genomic relationship information replaces pedigree relationship information, as in the animal model (Hayes *et al.* 2009).

The plant model focuses on the recombination phase in breeding of self-pollinating crops, and it is complementary to the traditional selfing process (Cornish 1990a,b) during which superior lines are identified for release as varieties, or for generating F_1_ hybrids. As a bonus, the model delivers a supply of selected S_2_ lines for further inbreeding and selection (Figure 1, Figure 2). The model reduces cycle time and increases response to selection while avoiding the pitfalls of crossing with inbred lines, such as slow cycles of selection, low effective population size (Cowling 2013), and the removal of additive genetic variance from the breeding population (Cornish 1990a,b).

We evaluated the model over two cycles of selection, which involved a single covariance per genetic component. The model could be extended to more cycles, sites, or traits, in which case a factor analytic model (Beeck *et al.* 2010; Cullis *et al.* 2010; Smith *et al.* 2001) would be best for modeling the covariances and assessing the impact of genotype by environment interaction. The model described here, where trials are “cycles” and data are combined across cycles, faces the same issues as BLUP analyses of multi-environment trial data (data from one cycle, combined across sites), and both are subject to genotype by environment interaction (Beeck *et al.* 2010; Cullis *et al.* 2010). This must be accounted for in future developments of the plant model. In any case, the model needs to fit separate variances for each source of variation in each trial.

Several improvements could be made to experimental methods, selection, and crossing design in this model. Replication was very low in cycle 1 (5%) and higher in cycle 2 (23%) (Table 2). The selection proportion of S_0_ plants in cycle 1 was low, with only 102/959 S_0_ plants promoted to cycle 2. This resulted in a major reduction in the number of cycle 1 parents that survived in pedigrees of cycle 2 (33/76). The crossing method of pair-wise crossing adopted in cycle 2 was a practical solution to include all 102 S_0_-derived lines in crossing, with replicates of each pair-wise cross performed between several S_1_ progeny. This may not be the optimum use of limited resources, and it may be better to relax selection pressure and include more S_0_ selections with fewer S_1_ parent plants per selection in crossing designs that minimize inbreeding and maximize potential genetic progress. We also lost several plants that did not survive the transplanting process and were included as missing values (Table S1, Table S2). Despite these imperfections, the mixed model analysis with pedigree relationship information provided a significant improvement over the base model and was further improved by including the additive covariance between cycles in the full model (Table 3).

We have shown that a relatively minor change in the practice of plant breeding (“crossing before selfing” rather than “selfing before crossing”), combined with BLUP selection methods from the animal model, has accelerated response to selection [1] in this selfing crop. Accuracy of selection in cycle 2 S_0_ progeny was high (>0.8). Generation interval was reduced to potentially 1 yr if crossing was performed in the contra-season. The method should help to retain additive genetic variance in breeding populations, which is lost when selection occurs during selfing and before crossing (Cornish 1990a,b). With further research, the method could be applied to traits measured on harvested seed of S_0_-derived lines (such as grain yield or grain quality). The approach could help to counter the low effective population size and low genetic diversity in many selfing and F_1_ hybrid crops (Kannenberg and Falk 1995; Duvick *et al.* 2004; Cowling 2013), but only if necessary steps are taken to avoid increases in inbreeding as a result of BLUP selection, such as optimal contribution selection (Henryon *et al.* 2015).

Our method also provides a continuous supply of preselected S_2_-derived lines for further selfing and selection (Figure 1, Figure 2). The model may be applied to genomic selection during early generations of selfing as proposed by Heslot *et al.* (2015), and it may be applied to incorporate new genetic diversity into elite breeding pools from wild and landrace types (Cowling *et al.* 2009; Falk 2010), as occurred in this project with ancestors, including elite cultivars from Australia and Europe, landraces from Greece and China, and wild-types (Beeck *et al.* 2006; 2008).

High levels of selfing in *P. sativum* made it possible for Mendel to discover the particulate nature of inheritance, and relationships derived from selfing in *P. sativum* contributed to the high accuracy of predicted breeding values of S_0_ progeny in this model for breeding self-pollinating crop plants.
